# Prevalence, Associated Risk Factors, and Identification of the Genera of Equine Strongyles in Horses and Donkeys in and Around Bishoftu, Ethiopia

**DOI:** 10.1155/vmi/3224113

**Published:** 2024-12-28

**Authors:** Asnakew Mulaw Berihun, Feyisa Bizu, Moges Maru, Seid Kassaw

**Affiliations:** Department of Pathobiology, College of Veterinary Medicine and Animal Sciences, University of Gondar, Gondar, Ethiopia

**Keywords:** donkey, genus's identification, horse, prevalence, risk factor, strongyle parasites

## Abstract

Equines play a significant role in the economy of the country. Besides their importance, equines face several challenges mainly helminth infections. Of these strongyle parasites are the most common, particularly in the study area. Thus, a cross-sectional study was conducted from February 2022 to July 2022 to determine the prevalence and associated risk factors and to identify the genera of equine strongyle parasites in and around Bishoftu. A total of 364 equines were randomly selected from five different areas and subjected to coprological examination using the floatation technique to detect strongyle-type eggs. A pooled faecal sample was cultured and subjected to modified Baerman techniques to identify the genus of strongyles. The overall prevalence of strongyle parasites was found to be 56.6% with an occurrence rate of 54.7% in horses and (65.2%) in donkeys. According to the present study, the two important genera identified were *Strongylus* and *Cyathostomum.* A higher prevalence of strongyle parasites was recorded in poor body condition (64%), adult (59.3%), female (70.7), and Kality (22%). Among the potential risk factors analyzed, the body condition score of the equines was significantly associated with strongyle infection (*χ*^2^ = 76.536 and *p* ≤ 0.001), while sex, species, age, and origins were not significantly associated with the prevalence of infection (*χ*^2^ = 2.644 and *p* = 0.266), (*χ*^2^ = 2.404 and *p* = 0.121), (*χ*^2^ = 0.609 and *p* = 0.435), and (*χ*^2^ = 3.205 and *p* = 0.524), respectively. In conclusion, strongyle parasites pose a major health problem for equines in the study area. They should focus on improving housing, increasing welfare awareness, implementing seasonal deworming, and enhancing the feeding practices of equines.

## 1. Introduction

Approximately 112.5 million domestic Equidae are found worldwide, comprising 44.3 million donkeys, 58.5 million horses, and the remaining mules [1]. The population of equines in Africa ranges from 17.6 million, including 11.6 million donkeys, 2.3 million mules, and 3.7 million horses [2]. In Ethiopia, the total equine population has reached 2.03 million horses, 7.43 million donkeys, and 0.4 million mules in sedentary areas [3]. Horses, donkeys, and mules are extensively utilized, particularly in rural socioeconomic activities. These animals serve various purposes across different sectors, including traction power, carting, recreation, festival participation, riding, and transportation [4].

However, these huge populations of equines cannot provide the expected benefit to the national and local economy due to several infectious and noninfectious diseases, providing unsatisfactory care management and giving significantly less attention, especially to donkeys [5]. Among the various factors contributing to the health deterioration of equine, parasites play a major role.

From parasitic health factors of equine, strongyle nematode parasites embrace the first place. The strongyles are nematode parasites found in the large intestine, specifically in the caecum and colon of equines. Strongyles of equines are categorized as large strangles and small strongyles (cytostome). The large strongyles comprise the three most important species found in equines *Strongylus vulgaris* (*S. vulgaris*), *Strongylus edentatus* (*S. edentatus*), and *Strongylus equinus* (*S. equinus*). *S. vulgaris*, *S. edentates*, and *S. equinus* are commonly known as the double-tooth strongyle, the toothless strongyle, and the triple-toothed strongyle, respectively. *S. vulgaris* is smaller than the other two large strongyle species [6]. Among the gastrointestinal nematodes of horses, large strongyle infections, particularly *S. vulgaris*, have long been considered one of the most common and pathogenic parasites affecting horses [7]. Strongylosis poses a serious problem in young horses raised on permanent horse pastures, although cases of severe disease may also occur in adult animals kept in suburban paddocks and subjected to overcrowding and poor management [8].

Strongylosis has been reported worldwide and affects nearly 90% of the horse population [9]. In Ethiopia, equines make significant contributions to the national economy, yet parasitic helminths are among the most common factors hindering their maximum utilization. These parasites cause varying degrees of damage depending on the species, nutritional status, and immune system of the equines, resulting in decreased performance and productivity, as well as increased morbidity and mortality [5]. The prevalence and types of internal parasites affecting equids are widespread, with equines being continually exposed throughout their lives [9]. *S. vulgaris* and *S. edentatus* are among the most common equine health problems caused by strongyle species in Ethiopia, with *S. equinus* being less common [10]. Although strongyle infections pose significant concerns for both the economy and equine health in Ethiopia, there is limited availability of recent data that shows the current status of strongyles, particularly in and around Bishoftu, Oromia, Ethiopia. Therefore, the objectives of this study were to determine the prevalence of strongyle parasites, to evaluate associated risk factors, and to identify the important genera of strongyles in horses and donkeys.

## 2. Materials and Methods

### 2.1. Study Area

**Figure 1:**
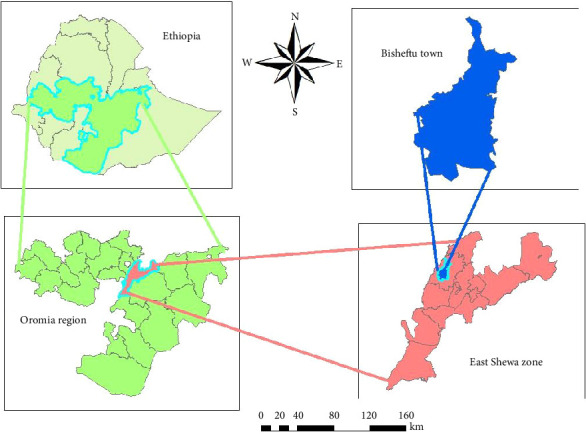
Study area map.

The study was conducted in and around Bishoftu, located approximately 47 km southeast of Addis Ababa. Bishoftu is situated at 9°N latitude and 40°E longitude, with an altitude of 1850 m above the sea level in the central highlands of Ethiopia (Figure 1). The region experiences a bimodal pattern of rainfall, with the main rainy season extending from June to September (accounting for 84% of the total rainfall) and a short rainy season from March to May, resulting in an average annual rainfall of 875 mm. The mean annual minimum and maximum temperatures are 14°C and 26°C, respectively, with an overall average of 20°C. The mean relative humidity is 61.3% [3].

### 2.2. Study Animals

The studied animals were 364 equines (298 horses and 66 donkeys) in and around Bishoftu town managed under a traditional extensive management system that originated from five localities (Kality, Dambi, Dalota, Bambogaya, and G/Gorba). The study encompassed both clinically suspected and healthy groups of donkeys and horses. The study animals were primarily used for traction, transport, and cart pulling, from which samples were obtained. During sampling, the animal species, sex, age, and body condition were recorded. The ages of the equines were determined from birth records of owner history and dentition characteristics. The body condition scoring was based on the criteria of donkey sanctuary and was classified into poor, moderate, and good. Faecal samples were collected exclusively from animals that had not been treated or dewormed in the 3 months preceding the study to ensure unbiased results.

### 2.3. Study Design and Sampling Techniques

A cross-sectional study was conducted from February 2022 to July 2022 to determine the prevalence of strongyle parasites in horses and donkeys, as well as to evaluate associated risk factors in and around Bishoftu. The study animal was conducted by using a simple random sampling method to examine the prevalence of equine (horses and donkeys) strongyles parasites. During the study, 364 equines (298 horses and 66 donkeys) were randomly selected based proportionality of their total numbers that were presented in and around Bishoftu town (Kality, Dambi, Dalota, Bambogaya, and G/Gorba). Five kebeles (peasant associations) were randomly selected using a sampling frame created from a complete list of kebeles in Bishoftu town. Similarly, the total population of each horses and donkeys in each kebele was determined, and the number of animals to be sampled was proportionally allocated based on their population in each kebele. Individual study animals were then randomly selected using a lottery system. The consent of their owners was obtained and conducted a brief interview. Faecal samples were collected directly from the rectum of the selected animals by the researchers to ensure sample quality and minimize bias.

### 2.4. Sample Size Determination

The required sample size of the study was determined by using the formula given by [11], with a 75.8% expected prevalence that was previously done, a 95% confidence level, and 5% desired absolute precision.

(1) $$\begin{array}{c} n=\frac{1.96^{2}\text{ Pexp}\left(1-\text{Pexp}\right)}{d^{2}}, \end{array}$$

where *n* is the sample size, *Z* (1.96) is the statistic corresponding to the level of confidence 95%, Pexp is the expected prevalence, and *d* is the desired absolute precision 5%. Therefore, based on the above formula, the desired sample size was 282 equines. However, the sample size was increased to 364, to increase the precision of the research and to obtain enough fecal egg outputs.

### 2.5. Study Methods

#### 2.5.1. Sample Collection Procedure and Processing

With gloved hands, approximately 10 g of fecal samples were directly collected from the rectum of restrained donkeys or horses in the clean sampling plastic bottle. Following collection, each sample was uniquely labeled with the animal's identification parameters, including species, age, sex, origin/site, and body condition score, and kept in the ice box. The collected samples were then packed and transported to the Addis Ababa University Veterinary Parasitology Laboratory for immediate examination of strongyle parasite eggs. If immediate processing is not possible, the sample was stored at +4°C in the refrigerator.

#### 2.5.2. Coprological Examination

The fecal samples were subjected to coprological examination for the fecal flotation technique. Approximately 3 g of feces were measured and placed into a glass beaker, followed by the addition of 40 mL of flotation fluid (magnesium sulfate). The mixture was continuously stirred using a glass rod, and the dissolved suspension was strained through a tea strainer into another beaker. The suspension was then transferred to a test tube until a meniscus formed at the top of the tube, and a cover slip was gently placed over the meniscus, allowing it to stand for 20 min [12]. The coverslip was removed and placed gently on a clean glass slide and examined under the microscope at low power magnification (10x) [12, 13].

#### 2.5.3. Identification of Strongyle at the Genus Level (Larvascopy)

It is important to recognize that the eggs of all equine strongyles are nearly identical and cannot be distinguished based on egg morphology alone, whether at the subfamily, genus, or species level. To achieve differentiation, the eggs can be cultured to hatch and develop to the L3 stage. This process involves a coproculture (fecal culture), followed by the Baermann technique and microscopic larval identification [14]. The modified Baermann technique was employed to isolate the third larval stage of nematode parasites from fecal cultures. First, a pooled fecal sample was collected from naturally infected horses that tested positive. The fecal culture of eggs to third-stage larvae was then carried out to differentiate the genus of equine strongyles. Ten g of fecal samples containing strongyle-type eggs were pooled, finely ground using a mortar and pestle, and cultured in a Petri dish with a small amount of water to moisten the sample. The samples were incubated at 27°C for 7 days and mixed periodically. The larvae were then recovered using the modified Baermann technique [15]. After collection, the third-stage larvae were mounted on slides, killed with Lugol's iodine, and identified under a microscope to the genus level based on morphological characteristics as described by [16, 17].

### 2.6. Data Management and Analysis

The data collected from the study were coded and entered in a Microsoft Excel spreadsheet 2007, and the statistical analysis was performed using SPSS Version 16 software packages. The chi-square test was used to assess the difference in the prevalence of strongyles nematode parasites among different variables such as equine species, sex, age, body condition, and origin. The percentage was used to calculate the prevalence rate of strongyles parasites. In all cases, 95% confidence interval (CI) and *p* < 0.05 were considered for a statistically significant difference. In the current study, the data distribution was a non-normal distribution.

## 3. Results

### 3.1. Results of Coprological Examinations

#### 3.1.1. Overall Prevalence

Based on the coprological analysis (egg morphology) (Supporting Information S1), the overall prevalence of strongyles nematode parasites of equine in the study area was found to be 56.6% (206/364) with a prevalence of 54.7% and 65.2% in horses and donkeys, respectively (Table 1).

#### 3.1.2. Risk Factors Analysis

The analysis highlights significant associations between risk factors such as sex (*χ*^2^ = 16.10 and *p* ≤ 0.001), age (*χ*^2^ = 14.78 and *p* ≤ 0.001), and body condition (*χ*^2^ = 8.60 and *p* = 0.013) with the prevalence of stronglyles infection in horse. However, prevalence, while origin shows no significant variation (*χ*^2^ = 3.32 and *p* = 0.506) in (Table 2).

The chi-square analysis revealed that there was no significant difference in the occurrence of strongyles parasitic infection among the equine species between horse and donkey (*χ*^2^ = 2.404 and *p* = 0.121) (Table 3). Regarding body condition of the equines, the highest relative prevalence was observed in poor (64%) than those in medium (54%) and good (50%). Prevalence in female (70.7%) animals was higher than in male (52%). The prevalence of strangles nematode parasites according to age group was 59.3% and 42.1% in adult and young equines, respectively. The prevalence of strongyles parasite was insignificantly varied between sexes (*χ*^2^ = 2.644 and *p* = 0.266) and age of the equines (*χ*^2^ = 0.609 and *p* = 0.435) but it was significantly varied among body conditions (*χ*^2^ = 76.536 and *p* ≤ 0.001) (Table 3).

**Figure 2:**
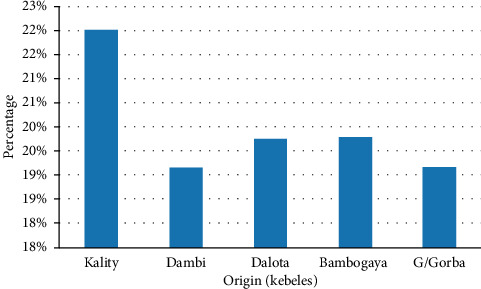
Prevalence of equine (horse and donkey) strongyle nematode parasites based on origin.

Among the five origins or sites study areas, the highest prevalence in horses and donkeys was revealed from Kality followed by Dalota, Bambogaya, Dambi, and G/Gorba, with the prevalence of 22%, 19.8%, 19.8%, 19.2%, and 19.2%, respectively. The study showed no statistically significant difference (*χ*^2^ = 3.205 and *p* = 0.524) between the study area and the prevalence of gastrointestinal parasites in both horses and mules (Figure 2).

### 3.2. Results of the Identified Genus of Equine Strongyles (Larascopy)

Larvascopy examination analysis revealed that the two genera of strongyles were identified by the morphological characteristics of third-stage larvae stages. These two important identified genera were *Cyathostomum* and *Strongylus*. *Cyathostomum* spp. and were characterized by short, small, and thin larvae with a thin and long tail, and *Strongylus* was long, large, and broad larvae with a short esophagus and well-defined gut cells with a long tail as shown in Supporting Information S2.

## 4. Discussion

The coprological examination done for the current study using floatation techniques revealed an overall prevalence of strongyle nematode parasites in and around Bishoftu town to be 56.6%. This finding was closely aligned with previous reports by [13], who reported a prevalence of 47.4% in horses and donkeys in and around Kombolcha town, Ethiopia. The current study finding's prevalence was lower than the reports of [18], who documented prevalence rates of 64.61%, 100%, 99%, and 100% for Menz keya gerbil district, East Shewa, Adaa, and Akaki of East Shewa, respectively. The variation in the prevalence reported might be due to variations in sample size and sampling time as seasonality affects the occurrence of the parasites, feeding practice, and deworming habits [19].

In the current finding, the prevalence of 65.2% and 54.7% were recorded in donkeys and horses, respectively, the difference being statically nonsignificant (*p* > 0.05). The present study result is relatively in line with the works of [20], who reported a prevalence of 70.8% in donkeys and 58.5% in horses from South Wollo. This finding was higher than the reports of [21], who reported a prevalence of 44.55% in donkeys and 48.2% in horses. However, the current study's results were lower than the reports of [7], which reported a prevalence of 87.8% in donkeys and 66.7% in horses from Gondar, the reports of [22], which revealed 100% and 99% prevalence in donkeys and horses, respectively, from East Shewa-Adaa, and another study that reported a higher prevalence of 99.15% in Sudan [23]. The higher prevalence in donkeys might be attributed to differences in feeding and deworming activities, while the lower prevalence in horses could be due to their predominantly being cart horses in the study area, which are less exposed and sometimes completely restricted from pasture and grazing. Furthermore, differences in prevalence in different areas might be attributed to variations in sampling areas, feeding systems, and accessibility to deworming and health services [20].

Regarding the body condition score of equines, the prevalence of strongyle parasites was 64%, 54%, and 50% in poor, medium, and good body-conditioned equines, respectively, and there was a statistically significant association (*p* < 0.05) between the existence of strongyle infection and the body condition score of equines. This finding is in agreement with previous reports by [5, 22], who reported a statistically significant association (*p* < 0.05) between the occurrence of strongyle infection and the body condition score of equines. However, there was no significant difference in the prevalence of strongyle parasites in the equines from different origins of the study area. This is similar to the findings of [23]. This may be due to the similarity in the agroecology of the study areas, the epidemiology of the parasites, and the management systems used for the equine species. In this study, there was no significant difference (*p* > 0.05) observed in the prevalence of the parasites between young and adult equines. The highest prevalence of strongyle parasite infestation was observed in adults (53%) compared with young equines (3.6%). It may be attributed to the declining body condition and immunity of adult equines compared with young ones, as older equines are more frequently exposed to strongyle parasites due to extensive work overload and undernourished conditions [24].

In the present study, the equine strongyle parasites genera *Srongylus* and *Cyathostomum* were identified. These findings were conducted based on characteristics of strongyle nematode larva morphology similar to other studies conducted worldwide as well as in Ethiopia. This finding supports previous reports of [25] in Cambridge [16], in Brazil [17], in the United States [26], and in Ethiopia. The current study revealed that equine strongyle infections are highly prevalent in the study area, indicating a significant health concern for equines. This highlights a critical gap in the control and prevention of parasitic diseases, suggesting that they are often overlooked or inadequately addressed. To reduce the prevalence of strongyle infections and improve the health and productivity of equines, it is essential to implement a strategic, programmatic approach to deworming. Moreover, this study has certain limitations, including the lack of an in-depth assessment of deworming practices, determination of infection levels, and evaluation of the efficacy of anthelmintic treatments. As such, it is recommended that future research builds upon this study as a baseline, conducting more comprehensive investigations to provide a more thorough, significant, and valuable contribution to equine owners, veterinarians, policymakers, and government agencies. These efforts will be crucial for developing effective interventions to control strongyle infections and improve overall equine health.

## 5. Conclusion and Recommendations

The current research underscores the vital role of equines as a means of transportation in rural and semiurban areas. However, strongyle parasites pose a significant threat to equine species and have a considerable economic impact. Equines often graze freely in pastures, exposing them to a high risk of parasitic diseases. Parasitized equines exhibit poor immunity and health status, leading to decreased working performance. Therefore, as a recommendation to control the burden of parasites, regular and strategic deworming programs using effective anthelmintics should be implemented consistently. Moreover, to obtain a clearer epidemiological picture, molecular characterization of parasites and assessment of anthelmintic resistance profiles should be conducted.

## Figures and Tables

**Table 1:** The overall prevalence of strongyle parasites in horse and donkey.

Equine species	Number of examined	Number of positive	Prevalence (%)	95% CI
Horse	298	163	54.7	49.0–60.3
Donkeys	66	43	65.2	53.7–76.6
Total	364	206	56.6	51.5–61.7

**Table 2:** The prevalence of strongyle parasites in horses in relation to various risk factors.

Risk factors		Number of equines examined	Number of positive	Prevalence (%)	95% CI	*X* ^2^	*p* value
BCS	Poor	120	77	63.68	56.85–70.52	16.10	0.013
	Medium	100	49	38.89	29.69–48.08	16.10	
	Good	78	35	66.51	60.20–72.82	41.78	

Age	Adult	215	143	24.10	14.90–33.30	41.78	0.001
	Young	83	20	64.17	55.59–72.75	8.62	

Sex	Male	190	121	49.00	39.20–58.80	8.62	0.001
	Female	108	42	44.87	33.83–55.91	8.62	

Origin	Kality	60	37	61.67	49.36–73.97	3.32	0.506
	Dambi	55	32	58.18	45.15–71.22	3.32	
	Dalota	58	33	56.90	44.15–69.64	3.32	
	Bambogaa	62	30	48.39	35.95–60.83	3.32	
	G/Gorba	63	31	49.21	36.86–61.55	3.32	

**Table 3:** Chi-square analysis of the association of different risk factors with the prevalence of strongyles parasite in horse and donkey.

Risk factor		Number of equines examined	Number of positive	Prevalence (%)	95% CI	*X* ^2^	*p* value
Species	Horse	298	163	54.7	49.0–60.3	2.404	0.121
	Donkey	66	43	65.2	53.7–76.6		

BCS:	Poor	139	89	64	56.1–72.0	76.536	0.001
	Medium	121	65	54	44.8–62.6		
	Good	104	52	50	40.4–59.6		

Age	Adult	307	182	59.3	53.8–64.8	0.609	0.435
	Young	57	24	42.1	23.9–54.9		

Sex	Male	275	143	52	46.1–57.9	2.644	0.266
	Female	89	63	70.7	61.3–80.2		

## Data Availability

The datasets for the current study are available from the corresponding author upon request.

## Supporting Information

Supporting Information S1: The eggs of equine strongyles (horse and donkey).

The eggs of equine strongyles were oval shaped with thin, smooth shell and contain Blastomeres as shown in the image.

Supporting Information S2: The larval stages of equine strongyles (genus: *Cyathostomin* and *Strongylus*).

The left side image showed the larva of genus *Cyathostomum* which characterized by short, small, and thin larvae with a thin and long tail, whereas the right side image showed the larva of genus *Strongylus* was long, large, and broad larvae with a short esophagus and well-defined gut cells with a long tail.
