# Supplementary material for: Prevalence, Associated Risk Factors, and Identification of the Genera of Equine Strongyles in Horses and Donkeys in and Around Bishoftu, Ethiopia
Source: Vet Med Int. 2024 Dec 28;2024:3224113. doi: 10.1155/vmi/3224113 (PMC11699984; doi:10.1155/vmi/3224113)
Supplement: Supporting Information — Additional supporting information can be found online in the Supporting Information section. [file 3224113.f1.zip › supplementary materials.docx]

**Supplementary materials**


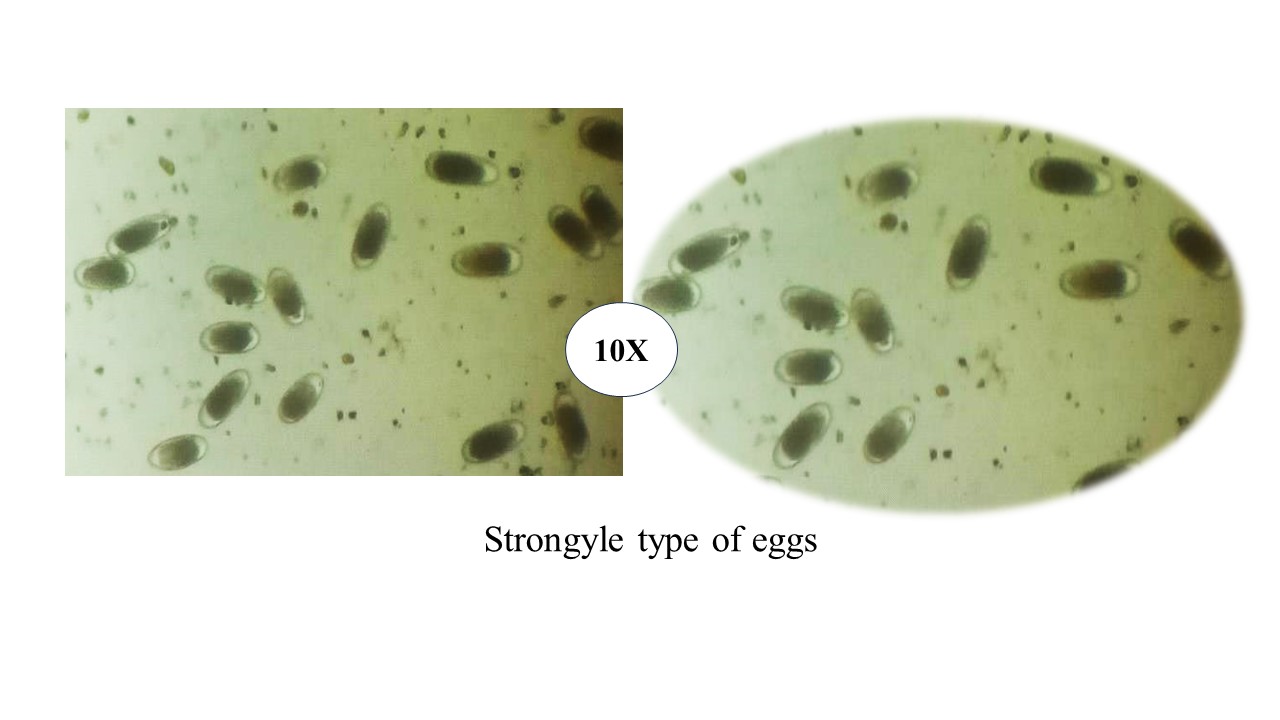
**Supplementary file S1:** The eggs of equine strongyles (horse and donkey)


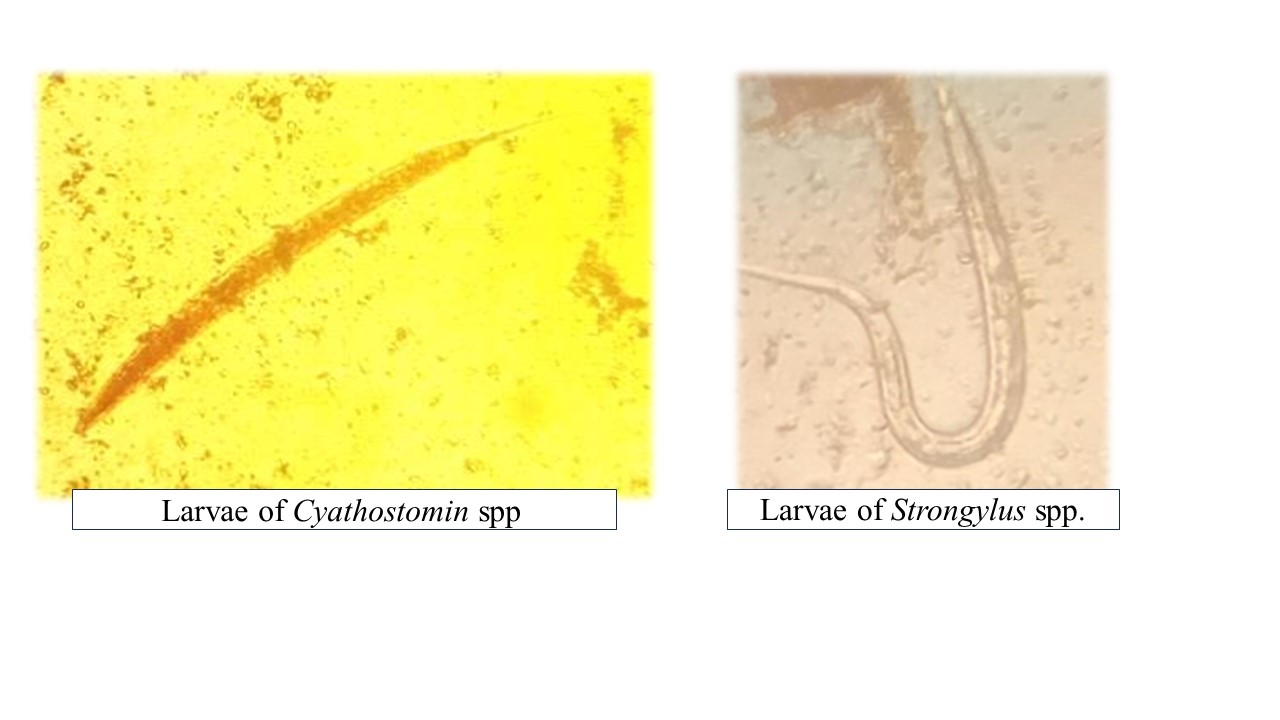


**Supplementary file S2:** The larval stages of equine strongyles
